# Molecular Cloning of *cpcU* and Heterodimeric Bilin Lyase Activity Analysis of CpcU and CpcS for Attachment of Phycocyanobilin to Cys-82 on the β-Subunit of Phycocyanin in *Arthrospira platensis* FACHB314

**DOI:** 10.3390/molecules21030357

**Published:** 2016-03-16

**Authors:** Fei Wu, Xiaonan Zang, Xuecheng Zhang, Ran Zhang, Xiaoyun Huang, Lulu Hou, Minjie Jiang, Chang Liu, Chunhong Pang

**Affiliations:** Key Laboratory of Marine Genetics and Breeding, Ministry of Education, Ocean University of China, Qingdao 266003, China; wufei0917@sina.cn (F.W.); xczhang@ouc.edu.cn (X.Z.); ikaibei@163.com (R.Z.); hxy764379760@163.com (X.H.); lu2014ouc@163.com (L.H.); jiangmjp@163.com (M.J.); 17864275931@163.com (C.L.); pangchunhong2014@126.com (C.P.)

**Keywords:** *Arthrospira platensis* FACHB314, *CpcU*, *CpcS*, site-directed mutation, fluorescence intensity

## Abstract

A new bilin lyase gene *cpcU* was cloned from *Arthrospira platensis* FACHB314 to study the assembly of the phycocyanin β-Subunit. Two recombinant plasmids, one contained the phycocyanobilin (PCB) producing genes (*hoxI* and *pcyA*), while the other contained the gene of the β-Subunit of phycobiliprotein (*cpcB*) and the lyase gene (*cpcU*, *cpcS*, or *cpcU/S*) were constructed and separately transferred into *Escherichia coli* in order to test the activities of relevant lyases for catalyzing PCB addition to CpcB during synthesizing fluorescent β-PC of *A. platensis* FACHB314. The fluorescence intensity examination showed that Cys-82 maybe the active site for the β-Subunit binding to PCBs and the attachment could be carried out by CpcU, CpcS, or co-expressed *cpcU/S* in *A. platensis* FACHB314.

## 1. Introduction

Phycobilisomes (PBSs) are multimeric highly-organized protein complexes that widely present in cyanobacteria and red algae, which can capture the 480 nm to 650 nm light energy and transmit the absorbed light to light system II [1,2]. PBSs are composed of colored phycobiliproteins (PBPs) and non-pigmented linker proteins. All major PBPs have a common subunit organization, in which α and β-Subunits form photometric heterodimers. The non-pigmented linker proteins are responsible for organize the PBPs into the PBSs and modulate their absorptions [2]. Cyanobacterial PBS is normally composed of two PBPs, phycocyanin (PC), and allophycocyanin (APC), while phycoerythrin (PE) appeared occasionally [1].

PBPs own their brilliant colors and light absorption properties to the presence of linear tetrapyrrole prosthetic groups, phycobilins, which are covalently attached to PBPs through thioether linkages to highly-conserved cysteine residues [3] (pp. 139–216). For PC, one phycocyanobilin (PCB) is attached to Cys-84 of α subunit (α-PC, CpcA), and two PCB chromophores are attached to Cys-82 and Cys-153 of the β-Subunit (β-PC, CpcB) [4]. Genetics and biochemical studies have shown that the products of the *cpcE* and *cpcF* genes form a heterodimeric lyase that specifically attaches PCB to Cys-82 of α-PC [5,6,7]. More recently, a group of four genes (*cpcS, cpcT, cpcU,* and *cpcV*) has been identified that codes for lyases attaching PCB to the β-subunits of CPC, and possibly allophycocyanin [8] (pp. 14–15). The possible function of CpcS has been rapidly characterized, thereafter. *In vitro* and in *Escherichia coli*, CpcS can catalyse chromophore attachment to all binding sites of APCs, to β-84 of CPCs and PECs, and even to some binding sites (α-84 and β-84) of CPEs [9,10,11]. These data indicated that S-type lyases are near-universal lyases for cysteine-84 binding sites in cyanobacterial phycobiliproteins. Based on amino acid sequence, CpcS can be classified into three clades. CpcS*-*I exists in cyanobacteria such as *Synechococcus* sp. PCC7002 and *Synechocystis* sp. PCC6803, which needs to form a heterodimer with CpcU to catalyse PCB attachment to β-PC and β-APC subunits [12]. CpcS*-*II exists in a variety of marine *Synechococcus* sp.; some of them produce PC with PEB chromophores [13]; and CpcS-III exists in cyanobacteria, such as *Anabaena* sp. PCC7120, which is able to ligate PCB to Cys-82 in a variety of PBPs as a single subunit [11,14].

*Arthrospira platensis* is a well-known economical Cyanobacteria, whose phycocyanin not only has biological activity on anti-oxidation, anti-tumor, anti-inflammation, and so on, but also contains the fluorescence property to be used as fluorescent tags, and possibly applied to the photodynamic therapy of cancers. In recent years, the research of expressing optically-active phycocyanin has made many achievements through genetic engineering, providing the foundation for establishment new photosynthesis system in transgenic plants. In our previous study, the gene *cpcS* was cloned from *A. platensis* FACHB314 [15], while it is uncertain whether it can work alone or work together with CpcU. Thus, we decided to clone the gene *cpcU* from *A. platensis* FACHB314 and study its function.

To study the catalytic function of the chromophore lyase, the heterologous host *Escherichia coli* has been used to express the optically-active phycocyanin. In *E. coli*, apophycocyanin (*cpcA, cpcB*), chromophore synthase (*hox1*, *pcyA*) and chromophore lyase (*cpcE, cpcF, cpcU, cpcS, cpcT*) are essential for forming a complete optically-active phycocyanin [9,11,14,16,17,18,19]. PCB synthesis in *E. coli* has been achieved by co-expressing the heme oxygenase 1 gene (*hoxI*) and PCB-ferredoxin oxidoreductase gene (*pcyA*) [18,20] (p. 508, pp. 459–462). Further, holo-α-PC was synthesized in *E. coli* by co-expressing *cpcA*, *cpcE/F*, *hoxI*, and *pcyA* [21]. Based on these, it seemed plausible that heterologous co-expression of various components in *E. coli* is an approach to understanding the assembly of PBS.

In this study, a bilin lyase gene, *cpcU* was first cloned from *A. platensis* FACHB314 and transformed into *E. coli* together with the apo-phycocyanin gene (*cpcB*) and chromophore synthase gene (*hox1*, *pcyA*) to test its function. This research has provided an experimental foundation for assembling the phycocyanin β-subunit and synthesizing optical phycocyanin in a heterologous host.

## 2. Results

### 2.1. Comparative Bioinformatics Analysis of cpcU

The complete coding sequence and the deduced amino acid sequence of CpcU are shown in the Supplementary Materials (Figure S1). The full length DNA of *cpcU* has 525 nucleotides with the GC content of 43.8%. The ORF, starting with the ATG at position 1 and ending with TAA at position 525, encodes a protein of 174 amino acids, with the theoretical isoelectric point of 5.30 and the predicted molecular mass of 19.2 kDa.

**Figure 1:**
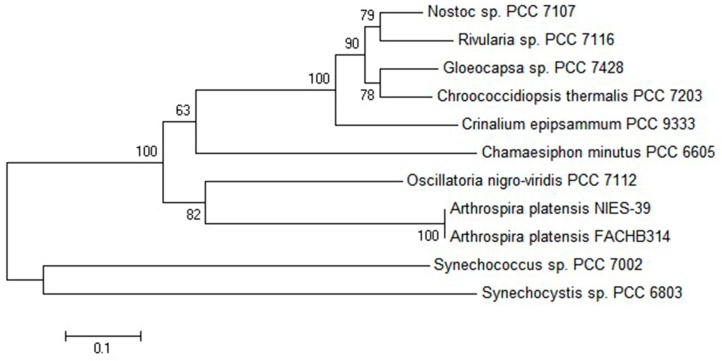
Phylogenetic analysis of CpcU amino acid sequences. Numbers at the nodes represent the bootstrap values. The evolutionary distance between the groups is indicated by the scale (0.1 = 10% differences). The sequences of CpeS taken from GenBank are as follows: *A.* *platensis* NIES-39 (NC016640.1), *Chamaesiphon minutus* PCC 6605 (CP003 600.1), *Oscillatoria nigro-viridis* PCC 7112 (CP00361 4.1), *Nostoc* sp. PCC 7107 (CP003548.1), *Rivularia* sp. PCC 7116 (CP003549.1), *Gloeocapsa* sp. PCC 7428 (CP0 03646.1), *Synechococcus* sp. PCC 7002 (EU145732.1), *Synechocystis* sp. PCC 6803 (CP003265.1), *Chroococcidiopsis* *thermalis* PCC 7203 (AFY87195.1), and *Crinalium epipsammum* PCC 9333 (NC0197531).

BLAST and homology analysis [22] revealed that the *cpcU* of *A. platensis* FACHB314 had high identity with the *cpeS* super family and contained the conserved motif of a chromophore lyase, including EFF, SAGKWFS, GKS, EER, PNLR, ASF, and SEIR. In the phylogenetic analysis [23] of CpeS from other algae (Figure 1), the CpcU of *A. platensis* FACHB314 clustered with *A. platensis* NIES-39 with the confidence of 100%, and was more distantly related to *Synechocystis* and *Synechococcus*.

### 2.2. The Construction of the Recombinant Expression Strains

Six expression vectors containing *cpcB*, *cpcB-cpcU*, *cpcB*(C82A)-*cpcU*, *cpcB*(C153A)-*cpcU*, *cpcB-cpcS*, and *cpcB-cpcU-cpcS*, respectively, were constructed and transformed into *E.* *coli* BL21 together with the vector pET-*hox1-pcyA* separately. Among them, the vectors containing *cpcB*, *cpcB-cpcU*, *cpcB-cpcS* and *cpcB-cpcU-cpcS* were used to study the function of CpcU. The vectors containing *cpcB*(C82A)-*cpcU*, *cpcB*(C153A)-*cpcU* were designed to mutate the amino acid Cys at the sites 82 and 153 of the β-subunit to Ala due to the different characteristics of Cys and Ala, which was used to examine the action site in the phycobiliprotein’s β-subunit catalyzed by CpcU in *A. platensis* FACHB314. According to the study of Shen in 2004, in *Synechococcus* sp. PCC 7002, the β-subunit of wild PC has two sites, the cysteine-82 and cysteine-153, which can bind PCB [8]. All the transformed strains were verified by PCR and sequencing to confirm that the inserted genes *cpcB*, *cpcU*, *cpcS*, and *hox1-pcyA* were present in the cells and the expression cassette was correct.

### 2.3. Expression of the Recombinant Proteins

**Figure 2:**
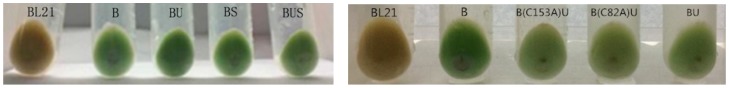
Color of cell pellets of transformed *E. coli* strains.

In comparison with the control (*E. coli* BL21) which was grey-white color, the recombinant stains showed different degrees of blue-green color (Figure 2). The result showed that the recombinant β-PC was expressed and attached with PCB successfully. Recombinant stain B also has the blue-green color, which suggests that the β-subunit of PC has autocatalytic activity for PCB attachment. The intensity of blue-green color of BU was higher than that of B, which may indicate that lyase CpcU has an activity in catalyzing attachment of PCB to β-PC. B(C153A)U and BU had nearly identical blue-green color, however, B(C82A)U was somewhat lighter in color compared to BU.

**Figure 3:**
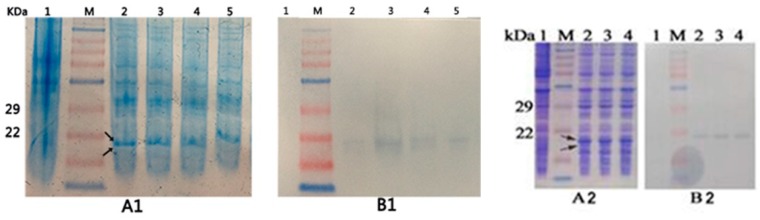
SDS-PAGE and Western blotting of *E. coli* BL21 and the recombinant strains. Line 1–5 in A1 and B1 is *E. coli* BL21, B, BU, BS, and BUS, respectively; Line 1–4 in A2 and B2 is *E. coli* BL21, BU, B(C82A)U and B(C153A)U, respectively. Arrows indicate the band of phycocyanin at about 18 KDa; A1, B1, A2 and B2 show that the blank *E. coli* BL21 has no immunoreactive band, whereas B, BU, BS, BUS, B(C82A)U and B(153A)U all have immunoreactive bands at about 18 kDa.

The SDS-PAGE gel (Figure 3) shows that the recombinant strains all have the phycocyanin band at about 18 kDa (arrows point to), which is not present in the control lane. The Western blotting (Figure 3) shows that the blank *E. coli* BL21 has no immunoreactive band, whereas the four recombinant strains (B, BU, BS, and BUS) and two mutant strains (B(C82A)U and B(153A)U) all have immunoreactive bands at about 18 kDa, indicating that phycocyanin was expressed in the recombinant *E*. *coli*.

### 2.4. Fluorescence Emission Spectra

**Figure 4:**
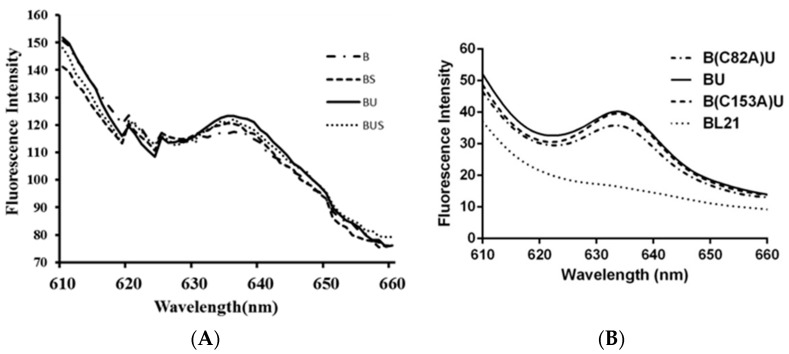
Fluorescence emission spectra of the recombinant strains (**A**) and mutant strains (**B**). All samples have been measured for three times.

The cell suspensions of recombinant strains were used to check the specific PC fluorescence emission spectra after excitation at 580 nm (Figure 4). All samples have been measured for three times and all of the data were analyzed by using statistical analysis method. After calculation of the fluorescence intensity of the unit concentration of phycocyanin (Table 1 and Table 2), the result showed that the spectrum of control *E. coli* BL21 was smooth, while the recombinant strains B, BU, BS, BUS, B(C82A)U, and B(153A)U all have a peak of fluorescence emission at about 633 nm, which verified the successful expression of the transformed genes and the attachment of PCB onto apo-PC. The recombinant strain B also showed fluorescence characteristics of PC, suggesting that β-PC has autocatalytic activity for the attachment with PCB. The recombinant strain BU shows a higher fluorescence intensity than that of the strain B (Figure 4A) and the difference is significant (*p* < 0.05). In addition, the fluorescence intensity of the strain BU seems higher than that of the recombinant strains BUS and BS (Figure 4A), but the difference is not significant (*p* > 0.05). BUS has the similar fluorescence intensity with BS. The result indicated that in *A. platensis* FACHB314, CpcU, and CpcS had the chromophore lyase function separately and did not show the coordination effect. B(C82A)U and B(153A)U both have a peak of fluorescence emission at about 633 nm; B(C82A)U almost has the same high fluorescence intensity with BU, while the peak of B(C82A)U is much lower than B(C153A)U (Figure 4B), which indicated that Cys-82 may be the active site of the β-Subunit catalyzed by CpcU to bind to phycocyanobilins in *A. platensis* FACHB314.

## 3. Discussion

**Figure 5:**
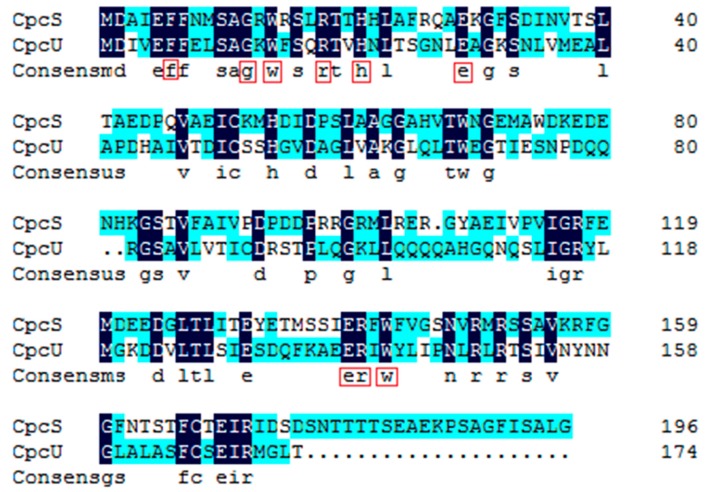
The sequence alignment of CpcS and CpcU of *A. platensis* FACHB314.

In this study, a chromophore bilin lyase gene *cpcU* of phycocyanin β-Subunit was first cloned from *A. platensis* FACHB314. The result of homology searches using the BLAST program show that CpcU is a relatively conserved protein in cyanobacteria, which has seven conserved domains including EFF, SAGKWFS, GKS, EER, PNLR, ASF, and SEIR (Supplementary Materials). CpcU and CpcS (FO818640.1) both are belong to CpeS superfamily by amino acid blasting, and Figure 5 shows that their similarity reaches to 29.8%. By multiple sequence alignment of CpcU with other cyanobacteria, two conserved amino acid tryptophan coding sites (14 and 142) were found, and the conservative tryptophan residues also exist in CpcS. We speculate that these two tryptophans may play an important role for chromophore lyase to exert its catalytic activity, and CpcU may have the similar chromophore lyase activity with the CpeS. To confirm this speculation, two recombinants were constructed to study and compare the function of the CpcU. In the recombinants, two polarity Cys at the 82 and 153 sites of phycocyanin β-Subunit were mutated to a non-polar-alanine (Ala) to study the working site of CpcU on phycocyanin β-Subunit. The results of SDS-PAGE and Western blotting showed that the mutant strain can also express phycocyanin, but the result of fluorescence spectra testing appeared that the fluorescence intensity of Cys^82^-mutant strains was lower than that of the non-mutated strain, while the fluorescence intensity of Cys^153^-mutant strain was almost equal with the non-mutated strain (Figure 4A). Based on these, we speculate that CpcU has an effect on the cysteine residue of the 82 site in phycocyanin β-Subunit, which was the same with the activity site of CpcS.

According to the report, whether in the cyanobacteria or in the heterologous host, once PCB was correctly ligated to apo-PC, its specific blue-green color and fluorescence spectrum should be detectable. Recombinant strains constructed in this research all appeared blue-green in color and present the specific fluorescence spectrum of PC. Recombinant strain B shows a relatively high intensity of fluorescence, suggesting that CpcB is able to attach PCB onto itself, spontaneously. Recombinant strains BU and BS show the higher fluorescence intensity than strain B, which suggests that both CpcU and CpcS have the function to attach PCB to β-PC. The fluorescence intensity of the recombinant strain BUS is between BU and BS, which indicates that there is no coordination effect between CpcU and CpcS in *A. platensis* FACHB314. Among the three strains of BU, BS, and BUS, BU shows the highest fluorescence intensity, which indicates that CpcU may work alone and play an important role in specific covalent attachment of PCB on the Cys-82 of β-PC. This result differs from those obtained in *Nostoc* sp. PCC7120. [11], in which only the product of CpeS was required for PCB addition to Cys-82 of β-PC. It is unclear why these two lyase subunits are required for some cyanobacteria, but not for others. According to the phylogenetic analyses, the *Nostoc* sp. PCC7120 belongs to a phylogenetically distinct subgroup of the CpcS-III. Whether organisms with CpcS-III arose by the loss of CpcU or organisms with *cpcS*-I gained *cpcU* by a gene duplication event is presently unclear.

According to the fluorescence spectra of recombinant strains, BU, BS, and BUS showed the same emission peak at 636 nm, with an 8 nm blue-shift from the native PC (λ_max_ = 644 nm). Such a blue-shift may be related to the change in chemical structure of PBPs. In wild-type strains, PC is composed of two subunits (αβ) but usually exists as a trimer (αβ)_3_ or a hexamer (αβ)_6_ [6,24]. In this study, only β-PC was expressed in *E. coli* and, thus, no integrated PC could be constructed. The absorption and emission peaks of the monomer are blue-shifted more than the trimmers, which is consistent with the report by MacColl *et al.* [25].

## 4. Materials and Methods

### 4.1. Strains and Plasmids

**Figure 6:**
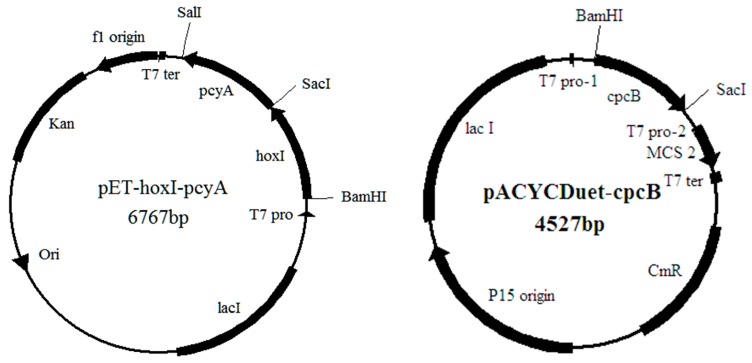

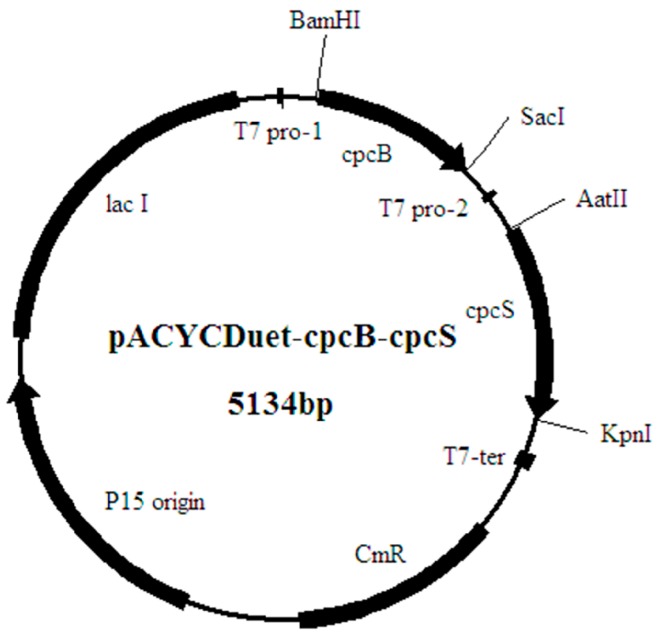
Plasmids of pET-*hoxI-pcyA*, pACYCDuet-*cpcB* and pACYCDuet-*cpcB-cpcS*.

The plasmid pET-*hox1-pcyA* was constructed to contain genes *hox1* and *pcyA* which were cloned from *A. platensis* FACHB314. These two genes could express enzymes catalyze heme into phycocyanobilins (PCB) in *E. coli*. The plasmid pACYCDuet-*cpcB* contains gene *cpcB*, encoding β-PC. The plasmid pACYCDuet-*cpcB-cpcS* contains genes *cpcB* and *cpcS*, among them, *cpcS* could express a chromophore lyase of *A. platensis* FACHB314 (Figure 6). All of the strains and plasmids used in this study are listed in Table 3.

### 4.2. Genomic DNA Isolation and Gene Cloning of cpcU

*A. platensis* FACHB314 was cultured in Zarrouk medium at 25 °C in our laboratory and its genomic DNA was extracted by using the Universal Genomic DNA Extraction kit (TaKaRa, Dalian, China).

According to the gene sequence analyze of *cpcU* in *Synechococcus* sp. PCC 7002 (GenBank No. CP000951.1), *Synechocystis* sp. PCC 6803 (GenBank No. CP003265.1), *Cyanothece* sp. PCC 7822 (GenBank No. CP002198.1), *Nostoc punctiforme* PCC 73102 (GenBank No. CP001037.1) and *A. platensis* NIES-39 (GenBank No. AP011615.1), primers (*cpcU*-F: 5′-GGAATTCCATATGATGGATATTGTCGAATTTTTTGAGTTG-3′, *cpcU*-R: 5′-GGAAGATCTTTACGTTAAAC CATGCGAATTTC -3′) were designed based on the conserved sequences. The restriction enzyme sites *NdeI* and *BglII* (underlined in the primer sequences) were incorporated at the 5’ ends of the primers to be used to digest and ligate the *cpcU* into the expression vector. The *cpcU* gene was amplified by PCR with the DNA of *A. platensis* FACHB314 as the template and the primers *cpcU*-F and *cpcU*-R. After being sequenced and blasted with the sequence in the GenBank, the PCR product was verified to be the *cpcU* preliminary.

### 4.3. Construction of the Recombinant Expression Vectors

The *cpcU* was double-digested with the restriction enzymes *NdeI* and *BglII* and inserted into the vector pACYCDuet-*cpcB* to produce the expression vector pACYCDuet-*cpcB-cpcU*. Then the vector pACYCDuet-*cpcB-cpcU* was transformed into the *E. coli* DH5α competent cell. After cultivation on the Luria-Bertani (LB) solid medium plate with chloramphenicol (34 μg∙mL^−1^), the positive clone was screened and verified by sequencing.

The plasmid pACYCDuet-*cpcB-cpcS* was extracted from recombinant strain BS using the High Pure Plasmid Extraction kit (Biomed, Beijing, China). The plasmid was digested by the restriction enzymes *AatII* and *KpnI*, and then ligated with the fragment *cpcU*, to yield the expression vector pACYCDuet-*cpcB-cpcU-cpcS*.

To study the ligation site of the β-Subunit with the PCB catalyzed by chromophore lyase CpcU, site-directed mutagenesis was used to mutate the amino acid Cys at the sites 82 and 153 of the β-subunit to Ala due to the different characteristics of Cys and Ala. By analyzing the preferred codons of *A. platensis FACHB314*, the codon for Ala should be GCT. So to mutate the amino acids Cys-82 and Cys-153 of *cpcB* to Ala, four primers were designed as follows: *cpcB*82-F5’-CGTCGTATGGCTGCTGCTTTGCG-3′, *cpcB*82-R5’-TGACATGGAAATCATCCTGCGCTATG-3′, *cpcB*153-F5’-GTATCACTCCTGGTGATGCTAGCGC-3′, and *cpcB*153-R5’-TTTGGCTTCAGAAATCGCTGGTTACTTTG-3′. Using the vector pACYCDuet-*cpcB-cpcU* as the template, the site-directed mutated DNA was obtained by PCR amplification using the Mutation Kit (TaKaRa). The mutated linear DNA was annularly connected using the DNA Blunting Kit (TaKaRa), and transformed into *E*. *coli* DH5α. The transformants were screened on Luria-Bertani (LB) plates containing 34 μg∙mL^−1^ of chloramphenicol (Cm) and verified by sequencing.

### 4.4. Plasmid Transformation and Protein Expression

Six recombinant expression plasmids (pACYCDuet-*cpcB*, pACYCDuet*-cpcB-cpcU*, pACYCDuet*-cpcB*(C82A)*-cpcU*, pACYCDuet*-cpcB*(C153A)*-cpcU*, pACYCDuet*-cpcB-cpcS*, and pACYCDuet*-cpcB-cpcU-cpcS*) were transformed into *E. coli* BL21 with pET-*hox1-pcyA* to produce the recombinant *E. coli* strains, including B, BU, B(C82A)U, B(C153A)U, BS, and BUS (Table 4). Positive colonies were selected on LB medium plates with the presence of 34 μg∙mL^−1^ chloramphenicol (Cm) and 100 μg∙mL^−1^ kanamycin (Km), and then confirmed by PCR amplification of the inserted genes.

For expression of the recombinant protein in the transformed strains, 2.5 mL starter culture cells were added into 250 mL LB medium with the antibiotics Cm and Km and shaken at 37 °C until the optical density at 600 nm (OD_600_) was 0.6. Production of the proteins was induced by the addition of 0.1 mM isopropyl-β-d-thiogalactoside (IPTG). Cells were incubated with shaking at 200 rpm at 37 °C for 3 h and the OD_600_ was measured again to ensure that the cell densities were the same before they were harvested by centrifugation at 4000 g for 25 min. Cell pellets were rinsed twice with 0.9% NaCl and then resuspended in 3 mL 0.1 M PBS buffer (pH 7.2). After ultrasonically lysed on ice for 6 min, cell suspensions were centrifuged at 12,000 *g* for 15 min and the supernatants were used for SDS-PAGE, Western blotting analysis and fluorescence emission spectra detection.

### 4.5. Recombinant Protein Analysis

Protein fractions were analyzed by polyacrylamide gel electrophoresis with the presence of SDS [10,22,23]. SDS-PAGE gel was made with 15% separation gel and 5% stacking gel (MDBio, Taiwan, China). Cells of the expression strains were lysed by ultrasonic disruption and centrifuged, and then the supernatant was boiled with 5× buffer before loading. The resolved proteins were visualized by staining with Coomassie Blue. Launch SensiAnsys was used for quantitative analysis of the percentage of the phycocyanin in the total protein. Then, the quantity of expressed recombinant phycocyanin was calculated by multiply the percentage of phycocyanin in the total protein and the total protein concentration. The total protein concentration was measured using a NANODROP 2000C spectrophotometer. Western blotting analysis was performed using the c-phycocyanin (C-PC) antibody as the primary antibody and peroxidase-conjugated goat anti-rabbit IgG as the secondary antibody. The hybridization bands in the nitrocellulose membrane were observed to confirm the expression of phycocyanin.

### 4.6. Fluorescence Emission Spectra

The fluorescence emission spectra for each sample by using a fluorescence spectrophotometer (HITACHI F-4600) excited at 580 nm. The quantity of each sample used for detection was 1 mL and all samples have been measured three times. The excitation and emission slit width was set at 10 nm with a scan speed of 1200 nm/min. To analyze the function of CpcU, the fluorescence intensity of the different strains should be compared. Thus, the fluorescence intensity per unit mass of recombinant phycocyanin was calculated by the ratio of the fluorescence intensity at the highest emission peak and the concentration of phycocyanin [13].

## 5. Conclusions

The present work has cloned a bilin lyase gene *cpcU* in *Arthrospira platensis* FACHB314 and the CpcU was confirmed to have the function to attach PCB to Cys-82 on β-PC. In our previous work, CpcS were found to attach PCB to Cys-82 on β-PC, and CpcT could attach PCB to Cys-153 on β-PC in *A. platensis* FACHB314. Compared with CpcT, the catalytic activity of CpcU and CpcS are weaker.

## Figures and Tables

**Table 1:** Quantitative and fluorescence intensity analysis of phycocyanin in B, BU, BS, and BUS.

Recombinant Strain	Total Protein Concentration (mg∙mL^−1^)	Phycocyanin (%)	Phycocyanin Concentration (mg∙mL^−1^)	Phycocyanin Fluorescence Intensity per Unit Mass
*E. coli*/B	42.07	1.98	0.83	117.55
*E. coli*/BU	37.96	2.63	1.11	123.39
*E. coli*/BS	43.28	2.13	0.92	120.68
*E. coli*/BUS	41.44	2.27	0.94	122.24

**Table 2:** Quantitative and fluorescence intensity analysis of phycocyanin in BU, B(C82)U, and B(C153)U.

Recombinant Strain	Total Protein Concentration (mg∙mL^−1^)	Phycocyanin (%)	Phycocyanin Concentration (mg∙mL^−1^)	Phycocyanin Fluorescence Intensity per Unit Mass
*E. coli*/BU	50.45	2.42	1.22	39.98
*E. coli*/B(C82)U	49.17	2.10	1.03	35.82
*E. coli*/B(C153)U	50.02	2.38	1.19	40.22

**Table 3:** Name and source of strains and plasmids used in this study.

Strain Source Application		
pMD18-T	TaKaRa (Dalian, China)	Cloning vector
pACYCDuet-1	Novagen (Germany)	Expression vector
*E*. *coli* DH5α	TaKaRa (Dalian, China)	Cloning strain
E. *coli* BL21	TaKaRa (Dalian, China)	Expression strain
pET-*hoxI-pcyA*	Our laboratory	Expression the chromophore synthase
pACYCDuet-*cpcB*	Our laboratory	Expression the β-PC
pACYCDuet-*cpcB-cpcS*	Our laboratory	Expression the β-PC and the chromophore lyase CpcS

**Table 4:** Designation of the recombinant strains.

Names of the Transformed	Expression Vectors	
*E. coli* Strains		
B	pACYCDuet-*cpcB*	pET-*hoxI-pcyA*
BU	pACYCDuet-*cpcB-cpcU*	pET-*hoxI-pcyA*
B(C82A)U	pACYCDuet-*cpcB*(C82A)-*cpcU*	pET-*hoxI-pcyA*
B(C153A)U	pACYCDuet-*cpcB*(C153A)-*cpcU*	pET-*hoxI-pcyA*
BS	pACYCDuet-*cpcB-cpcS*	pET-*hoxI-pcyA*
BUS	pACYCDuet-*cpcB-cpcU-cpcS*	pET-*hoxI-pcyA*

## Supplementary Materials

Supplementary materials can be accessed at: http://www.mdpi.com/1420-3049/21/3/357/s1.
