# Supplementary material for: Molecular Cloning of cpcU and Heterodimeric Bilin Lyase Activity Analysis of CpcU and CpcS for Attachment of Phycocyanobilin to Cys-82 on the β-Subunit of Phycocyanin in Arthrospira platensis FACHB314
Source: Molecules. 2016 Mar 16;21(3):357. doi: 10.3390/molecules21030357 (PMC6273044; doi:10.3390/molecules21030357)
Supplement: Supplementary file 1 [file molecules-21-00357-s001.pdf]

Molecules **2016**, *21*, 357; doi:10.3390/molecules21030357

Supplementary Materials: Molecular Cloning of *cpcU* and Heterodimeric Bilin Lyase Activity Analysis of CpcU and CpcS for Attachment of Phycocyanobilin to Cys-82 on the β Subunit of Phycocyanin in *Arthrospira platensis* FACHB314

Fei Wu, Xiaonan Zang, Xuecheng Zhang, Ran Zhang, Xiaoyun Huang, Lulu Hou, Minjie Jiang, Chang Liu and Chunhong Pang

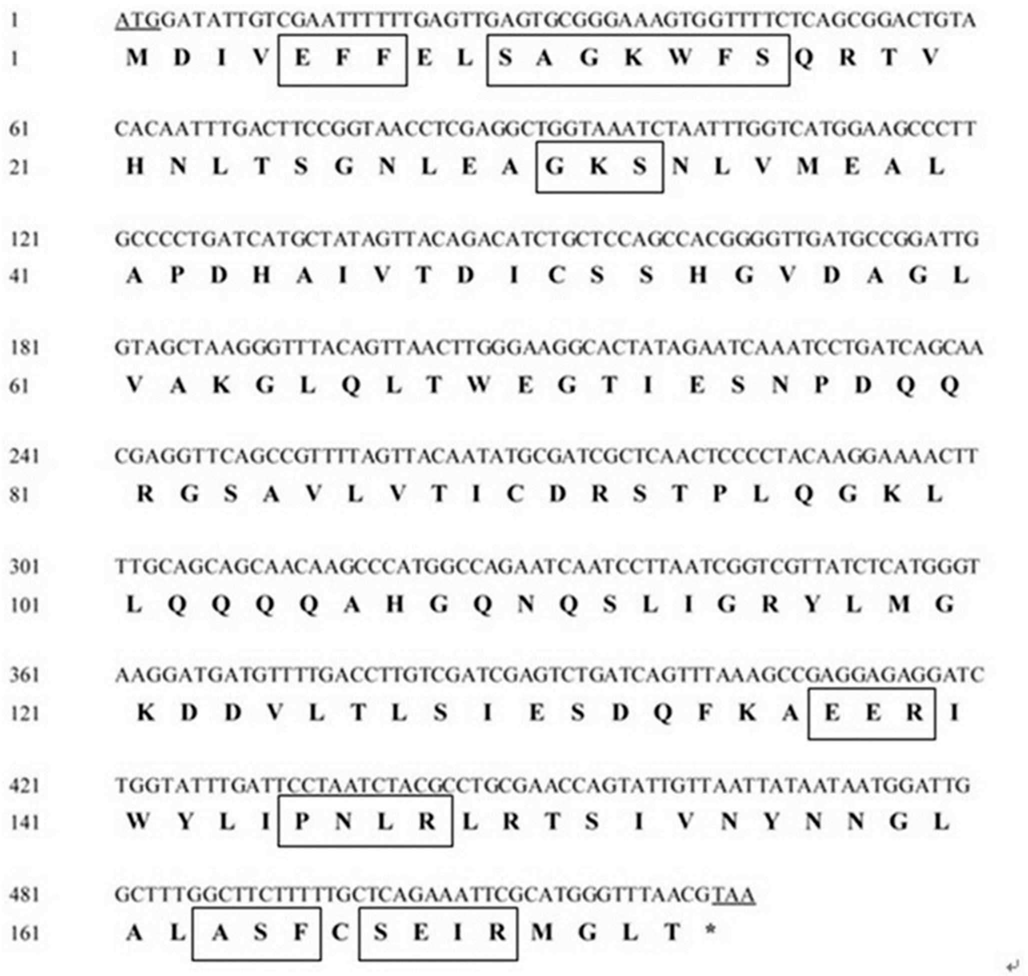

Figure S1. Nucleotide and amino acid sequence of CpcU. Underline indicates the ATG start codon and the TAA stop codon, and boxes show the conserved active sites.
